# Association of free thyroxine with obstructive lung pattern in euthyroid middle-aged subjects: A population-based study

**DOI:** 10.1371/journal.pone.0270126

**Published:** 2022-07-22

**Authors:** Hye Jeong Kim, Sang Joon Park, Hyeong Kyu Park, Dong Won Byun, Kyoil Suh, Myung Hi Yoo

**Affiliations:** 1 Division of Endocrinology and Metabolism, Department of Internal Medicine, Soonchunhyang University Hospital, Soonchunhyang University College of Medicine, Seoul, Korea; 2 Elim Thyroid Clinic, Seoul, Korea; Seoul National University College of Medicine, REPUBLIC OF KOREA

## Abstract

**Background:**

The association between thyroid hormone levels and pulmonary function in euthyroid population is still unclear. We aimed to examine the relationship between thyroid function and lung function in a large cohort study of euthyroid subjects.

**Methods:**

We analyzed biochemical and spirometry data from a nationwide, population-based, cross-sectional survey (KNHANES VI). A total of 1,261 middle-aged participants aged 45–65 years with spirometry tests and normal thyroid function were included in this study. The subjects were grouped according to free thyroxine (fT4) (ng/dL) quartiles (Q1, 0.89–1.09; Q2, 1.10–1.19; Q3, 1.20–1.30; Q4, 1.31–1.76). Obstructive lung pattern was defined as forced expiratory volume in one second (FEV_1_)/forced vital capacity (FVC) <0.7. The probability of obstructive lung patterns according to fT4 quartiles was assessed using logistic regression models, adjusted for potential confounders.

**Results:**

Overall, 10.9% of the subjects had an obstructive lung pattern. The mean fT4 levels were significantly higher in those with obstructive lung pattern than in those with normal lung function (1.26 vs. 1.20 ng/dL, p<0.001). The proportion of participants with obstructive lung pattern increased across the fT4 quartile categories (p<0.001). With the Q1 group as reference, the multivariate-adjusted odds ratios (95% confidence intervals) for obstructive lung pattern in the Q3 and Q4 groups were 2.875 (1.265–6.535) and 2.970 (1.287–6.854), respectively, even after adjusting for confounding variables.

**Conclusion:**

High fT4 levels are an independent predictor of obstructive lung pattern in euthyroid middle-aged subjects. Further prospective studies are needed to confirm these findings.

## Introduction

Thyroid dysfunction can lead to pulmonary dysfunction by causing respiratory muscle dysfunction or changes in the ventilator drive. Hyperthyroidism is associated symptoms of dyspnea on exertion, probably due to respiratory muscle weakness and a less-than-normal increase in cardiac output, and rarely accompanied with pulmonary hypertension [1]. In hypothyroidism, pulmonary dysfunction may be caused by pleural effusion, impaired respiratory muscle function, diminished ventilator drive, or sleep apnea [2]. In previous studies, hyperthyroid patients had a lower lung function than in euthyroid controls [3,4]. On the other hand, some studies have reported the lower lung function in patients with hypothyroidism than euthyroid controls [5,6], while other studies showed no association between hypothyroidism and lung function [7,8]. Ittermann *et al*. has been suggested that thyroid dysfunction is not associated with lung function in the general population [9], but little is known about the association between thyroid function and lung function in the general population.

Poor lung function is mainly determined by estimating the forced vital capacity (FVC) and forced expiratory volume in one second (FEV_1_) [10]. Clinically, a decrease of <0.7 in the spirometry parameter FEV_1_/FVC ratio indicates an obstructive pattern of poor lung function [10,11]. Chronic obstructive lung disease (COPD), a representative obstructive lung disease, is characterized by an incompletely reversible and usually progressive persistent airflow limitation that is associated with chronic inflammation [11]. Comorbid conditions, including cardiovascular disease, diabetes and hypertension, are highly prevalent among patients with COPD [12]. COPD can be seen as a pulmonary component of chronic systemic inflammatory syndrome [13]. Alterations in thyroid function are commonly observed in patients with COPD [7]. To date, several small-patient studies have investigated the association between thyroid function and lung function in COPD [7,14–16], but the results have been inconsistent. Furthermore, it is unclear whether the significant findings between thyroid function and lung function found in patient studies can be extended to generally healthy individuals.

The aim of the present study was to investigate whether thyroid hormones are related to obstructive lung pattern in euthyroid subjects in a large cohort.

## Subjects and methods

### Study population

This study used data from the Korea National Health and Nutrition Examination Survey (KNHANES) VI (2013–2015). The KNHANES is a nationwide, cross-sectional survey conducted by the Korean Centers for Disease Control and Prevention (KCDC) to assess the health and nutritional status of the Korean population [17]. The study subjects are selected using stratified, multistage cluster sampling of the population and housing census data. Among the participants, approximately 2400 individuals (1/3 sample of the participants aged ≥10 years) are selected for laboratory tests of serum thyroid-stimulating hormone (TSH) and free thyroxine (fT4) using stratified subsampling according to sex and age in each year [17].

**Fig 1:**
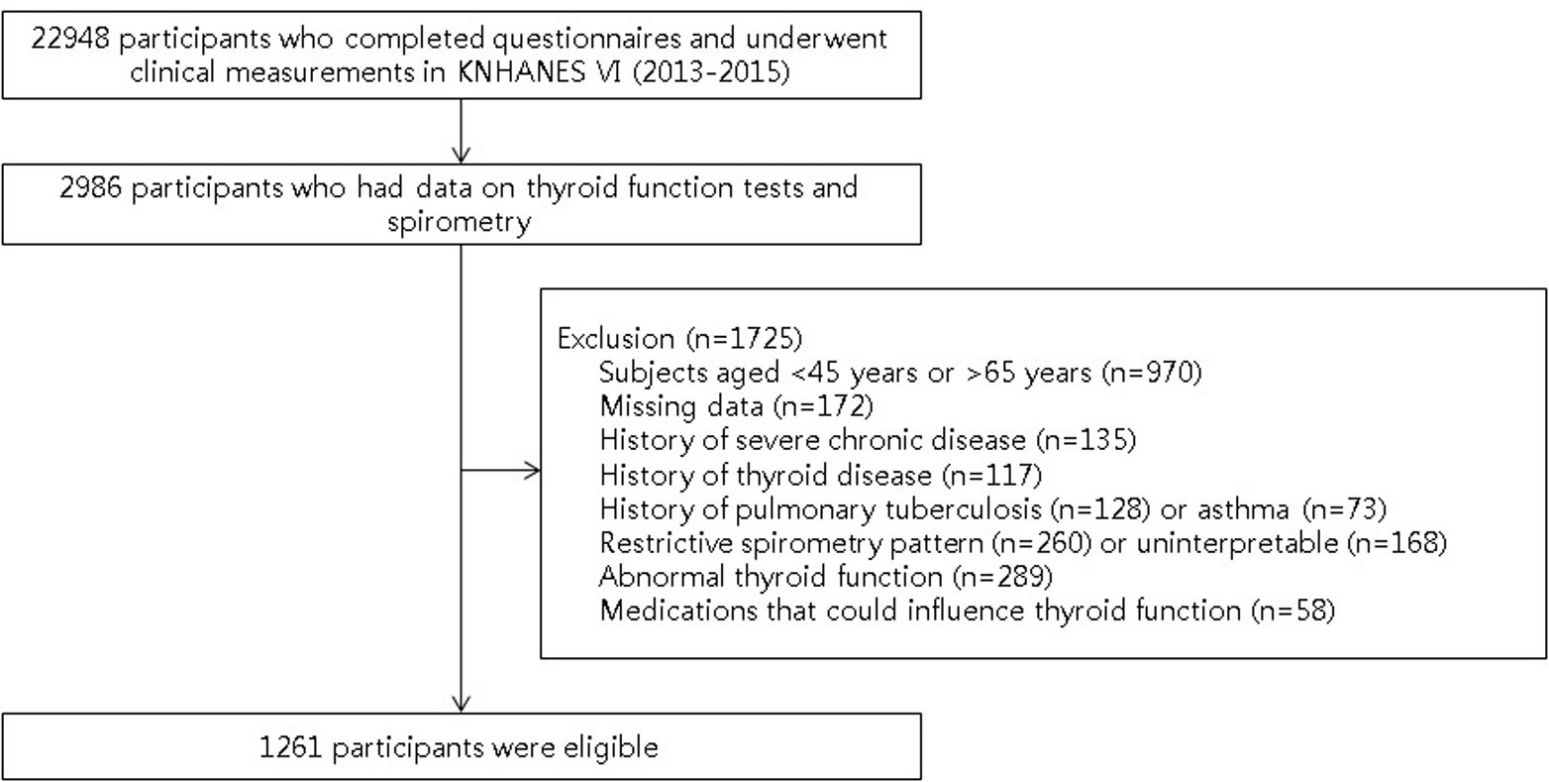
Flow chart of the study population. KNHANES, Korean National Health and Nutrition Examination Survey.

The current study evaluated 2,986 participants along with the results of their thyroid function tests and spirometry data. Among them, subjects were excluded due to (1) participants aged <45 years or >65 years (n = 970); (2) missing data (questionnaires about occupation, smoking, alcohol or exercise; and history of cancer, chronic kidney disease, liver cirrhosis, thyroid disease, pulmonary tuberculosis or asthma) (n = 172); (3) history of severe chronic disease, such as any type of cancer, chronic kidney disease or liver cirrhosis (n = 135); (4) history of thyroid disease (n = 117); (5) history of pulmonary tuberculosis (n = 128) or asthma (n = 73); (6) restrictive spirometry pattern (n = 260) or uninterpretable spirometry data (n = 168); (7) abnormal thyroid function (TSH and/or fT4 levels below or above the reference range) (n = 289); (8) medication that could influence thyroid function (n = 58). Several subjects met more than two exclusion criteria. Finally, 1,261 subjects were included in the analysis (Fig 1).

All subjects participated voluntarily in the survey and written informed consent was obtained from all individuals. All survey protocols were approved by the Institutional Review Board of the KCDC (approval numbers: 2013-07CON-03-4C, 2013-12EXP-03-5C, and 2015-01-02-6C).

### Clinical and anthropometric measurements

Occupation and health-related behaviors, including smoking, alcohol consumption and walking activity were assessed using a self-reported questionnaire. Occupation was classified into four categories: clerical (sales and service, clerical staff, administration and specialist), manual (agriculture, forestry, fishery, and manual labor), technical (engineering, assembling, and technical work), and unemployed (no job, students, and housewives) [18]. Smoking status was categorized as current, former, or never smoker. Smokers were defined as those with a self-reported history of smoking >100 cigarettes in their lifetime [19]. Current smokers were classified into three categories according to the daily amount of cigarettes smoked: heavy (≥21 cigarettes/day), moderate (11–20 cigarettes/day), and light (≤10 cigarettes/day) [20]. Former smokers reported that they only had a past history of smoking. Alcohol consumption was classified as excessive (>21 drinks/week in men and >14 drinks/week in women) [21], moderate (≤21 drinks/week in men and ≤14 drinks/week in women), or never drinkers [22]. Walking activity was categorized as either active or inactive. Active was defined as walking for at least 5 days per week and at least 10 minutes per day [23].

A physical examination was performed by trained medical staff following standardized procedures. Height was measured using a stadiometer (SECA 225; SECA GmbH, Hamburg, Germany) to the nearest 0.1 cm with the subject in the standing position. Body weight was measured with the participant in a light gown and bare feet using a digital scale (GL-6000-20; G-tech, Korea) to the nearest 0.1 kg, and body mass index was calculated by dividing the weight by the square of the height (kg/m^2^). Using a tape measure (SECA 200; SECA GmbH), waist circumference was measured to the nearest 0.1 cm in a horizontal plane at the level of the midpoint between the iliac crest and the costal margin at the end of expiration [24]. BP was measured on the right arm using a standard mercury sphygmomanometer (Baumanometer Desk Model 0320; WA Baum Co., Copiague, NY, USA) with the subjects in a sitting position. All BP measurements were taken in triplicate, and the mean of the second and third measured values was used in the analyses [24].

### Measurement of thyroid function

Blood samples were processed and transported to the central certified laboratory, and analyzed within 24 h. For thyroid function tests, approximately 15 mL of blood was collected from each participant. After separating the serum within 30 min, the samples were sent to a certified central laboratory and analyzed. Serum TSH and fT4 levels were measured using an electrochemiluminescence immunoassay (Roche Diagnostics, Mannheim, Germany). Serum TSH levels were assessed using an E-TSH kit (Roche Diagnostics). This study used the TSH reference range of 0.62–6.68 mIU/L for the Korean population [17]. Serum fT4 levels were assessed using an E-Free T4 kit (Roche Diagnostics), with a laboratory reference range of 0.89–1.76 ng/dL.

### Measurement of lung function

Lung function was measured using a dry rolling seal spirometer (Sensor Medics, Yorba Linda, CA, USA) according to the American Thoracic Society/European Respiratory Society criteria for standardization [25]. Spirometry data obtained on-site by clinical technicians were transferred to an Internet review center for processing, and the data were carefully examined and compared against criteria metrics for acceptability, reproducibility, and quality control [18].

### Definitions

Euthyroidism was defined as serum TSH (reference range, 0.62–6.68 mIU/L) [17] and fT4 (reference range, 0.89–1.76 ng/dL) levels within the normal reference ranges.

Obstructive lung pattern was defined as an FEV_1_/FVC <0.7 [10,11].

### Statistical analysis

Weighted sample values were used for analysis to reflect the stratified multistage probability sampling design of KNHANES VI. Continuous variables are reported as means (standard error), and categorical variables are presented as weighted percentages (%). The demographic and biochemical characteristics of the study population with respect to obstructive lung pattern were compared using general linear model for continuous variables and the chi-square test for categorical variables [26]. Spirometry data according to TSH quartiles or fT4 quartiles were compared using general linear model or chi-square test [26]. Complex samples logistic regression analyses were used to determine the risk of obstructive lung function patterns based on fT4 quartiles [27]. The results were expressed as odds ratios (ORs) with 95% confidence intervals (CIs). All p values and 95% CI for OR were corrected using Bonferroni’s method due to multiple testing. Additional adjustments were made for confounding variables, such as age, sex, occupation, smoking, alcohol consumption, walking activity, body mass index, glycated hemoglobin, and TSH.

All statistical analyses were performed using SPSS Statistics version 26.0 (IBM Corp., Chicago, IL, USA). All tests were two sided, and a p value of <0.05 was considered statistically significant.

## Results

In the cohort, men were 54.3%, and the mean subject age was 54.53 (0.18) years. Obstructive lung patterns were prevalent in 10.9% (n = 141) of the cohort. The anthropometric, clinical, and laboratory data of the subjects according to obstructive lung pattern are listed in Table 1.

**Table 1:** Baseline characteristics of study population by the status of obstructive lung pattern.

Variables	Obstructive lung pattern			Overall (N = 1,261)
	No (n = 1,120, 89.1%)	Yes (n = 141, 10.9%)	p value	
**Male (%)**	50.1%	88.7%	<0.001	54.3%
**Age (years)**	53.98 (0.21)	57.36 (0.56)	<0.001	54.34 (0.20)
**Occupation**^**a**^ **Clerical** **Manual** **Technical** **Unemployed**	38.1% 16.2% 19.3% 26.5%	30.0% 24.5% 17.4% 28.2%	0.129	37.2% 17.1% 19.1% 26.7%
**Smoking** **Current**^**b**^ **Amount of daily smoking** **Heavy** **Moderate** **Light** **Former** **Never**	22.9% 2.6% 12.8% 7.5% 22.8% 54.3%	41.2% 7.1% 24.2% 9.9% 41.5% 17.3%	<0.001	24.9% 3.1% 14.0% 7.8% 24.9% 50.3%
**Alcohol consumption**^**c**^ **Excessive** **Moderate** **Never**	14.5% 60.0% 25.5%	30.3% 50.2% 19.5%	<0.001	16.2% 58.9% 24.9%
**Walking activity** **Active** **Inactive**	51.8% 48.2%	41.7% 58.3%	0.045	50.7% 49.3%
**BMI (kg/m**^**2**^)^**d**^	24.15 (0.09)	24.04 (0.23)	0.665	24.14 (0.08)
**<18.5 kg/m**^**2**^ **18.5~24.9 kg/m**^**2**^ **25~29.9 kg/m**^**2**^ **≥30 kg/m**^**2**^	1.2% 63.2% 32.6% 3.0%	1.2% 66.9% 29.9% 1.9%	0.797	1.2% 63.6% 32.3% 2.8%
**Waist circumference (cm)** **Male** **Female**	85.28 (0.37) 79.53 (0.42)	86.39 (0.74) 79.43 (2.12)	0.188 0.963	85.48 (0.33) 79.52 (0.41)
**Systolic BP (mmHg)**	119.65 (0.58)	120.89 (1.43)	0.431	119.78 (0.53)
**Diastolic BP (mmHg)**	77.78 (0.33)	78.58 (0.89)	0.401	77.86 (0.30)
**Total cholesterol (mg/dL)**	197.21 (1.20)	190.69 (3.51)	0.075	196.50 (1.16)
**HDL cholesterol (mg/dL)** **Male** **Female**	47.31 (0.62) 52.92 (0.61)	47.38 (1.06) 51.22 (2.74)	0.951 0.548	47.32 (0.55) 52.87 (0.60)
**Triglycerides (mg/dL)**	153.47 (4.62)	159.13 (8.31)	0.546	154.09 (4.24)
**Fasting glucose (mg/dL)**	101.54 (0.67)	105.95 (2.88)	0.132	102.02 (0.69)
**Glycated hemoglobin (%)**	5.86 (0.32)	5.99 (0.11)	0.220	5.88 (0.03)
**TSH (mIU/L)** **Unadjusted** **Smoking-adjusted**	2.48 (0.04) 2.47 (0.04)	2.26 (0.10) 2.40 (0.10)	0.047 0.520	2.46 (0.04)
**fT4 (ng/dL)** **Unadjusted** **Smoking-adjusted**	1.20 (0.01) 1.20 (0.01)	1.26 (0.01) 1.25 (0.01)	<0.001 <0.001	1.21 (0.01)

BMI, body mass index; BP, blood pressure; HDL, high-density lipoprotein; LDL, low-density lipoprotein; TSH, thyroid-stimulating hormone; fT4, free thyroxine.

^a^Occupation classification: Clerical (sales and service, clerical staff, administration and specialist), manual (agriculture, forestry, fishery, and manual labor), technical (engineering, assembling, and technical work), and unemployed (no job, students, and housewives);

^b^Current smoker category: Heavy (≥21 cigarettes/day), moderate (11–20 cigarettes/day), and light (≤10 cigarettes/day);

^c^Alcohol consumption classification: Excessive (>21 drinks/week in men and >14 drinks/week in women), moderate (≤21 drinks/week in men and ≤14 drinks/week in women), and never drinkers;

^d^BMI category according to the WHO classification: Underweight (<18.5 kg/m^2^), normal weight (18.5–24.9 kg/m^2^), overweight (25–29.9 kg/m^2^), and obese (≥30 kg/m2).

Data are presented as means (standard error) or weighted percentages as appropriate for the variable. Demographic and biochemical characteristics of the study population with respect to the obstructive lung pattern were compared using general linear model for continuous variables and chi-square test for categorical variables.

Compared with normal lung function group, the obstructive lung pattern group included more men, older subjects, and current or former smokers, and were more likely to consume alcohol and be inactive. They had lower levels of TSH and higher levels of fT4. However, there was no significant difference between the groups in smoking-adjusted mean TSH levels.

We further investigated the spirometry data according to fT4 (ng/dL) quartiles: Q1, 0.89 to 1.09 (n = 294, 23.4%); Q2, 1.01 to 1.19 (n = 321, 25.5%); Q3, 1.20 to 1.30 (n = 317, 25.2%); and Q4, 1.31 to 1.76 (n = 329, 25.9%) (Table 2).

**Table 2:** Comparison of spirometry data according to free thyroxine quartiles.

Variables	fT4 quartile				p value
	Q1 (0.89–1.09 ng/dL) (n = 294, 23.4%)	Q2 (1.10–1.19 ng/dL) (n = 321, 25.5%)	Q3 (1.20–1.30 ng/dL) (n = 317, 25.2%)	Q4 (1.31–1.76 ng/dL) (n = 329, 25.9%)	
**Spirometry parameters**					
**FVC (L)**	3.63 (0.05)	3.73 (0.06)	3.79 (0.05)	3.98 (0.05)	<0.001
**FVC (%, predicted)**	96.08 (0.62)	95.71 (0.69)	95.47 (0.58)	95.32 (0.60)	0.840
**FEV1 (L)**	2.84 (0.03)	2.89 (0.04)	2.92 (0.04)	3.06 (0.04)	0.001
**FEV**_**1**_ **(%, predicted)**	95.69 (0.63)	94.35 (0.71)	93.42 (0.67)	92.78 (0.71)	0.012
**FEV**_**1**_**/FVC**	0.786 (0.003)	0.779 (0.004)	0.775 (0.004)	0.769 (0.004)	0.004
**FEF**_**25-75**_ **(L/sec)**	2.78(0.05)	2.74 (0.05)	2.74 (0.06)	2.81 (0.07)	0.898
**Obstructive lung pattern (%)**	4.9%	9.3%	13.0%	15.9%	<0.001

fT4, free thyroxine; FVC, forced vital capacity; FEV_1_, forced expiratory volume in one second; FEF_25-75_, forced expiratory flow, mid-expiratory phase.

Data are presented as means (standard error) or weighted percentages as appropriate for the variable. Spirometry data of the study population with respect to the free thyroxine quartiles were compared using general linear model for continuous variables and chi-square test for categorical variables.

FVC (L), FEV_1_ (L), FEV_1_ (%, predicted) and FEV_1_/FVC levels was significantly different among the groups. As the fT4 quartile categories increased, the FEV_1_ (%, predicted) and FEV_1_/FVC levels decreased. The proportion of participants with obstructive lung pattern increased across the fT4 quartile categories (p<0.001). There were no significant differences among the groups with respect to FVC (%, predicted) and forced expiratory flow, mid-expiratory phase (FEF_25-75_).

**Fig 2:**
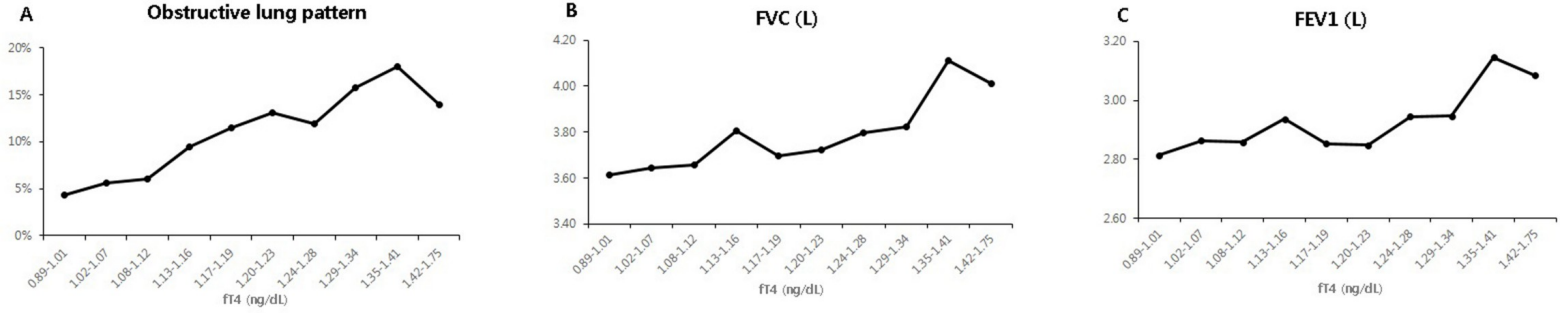
Relationship between free thyroxine (fT4) and lung function. The obstructive lung pattern (A), forced vital capacity (FVC) (B) and forced expiratory volume in one second (FEV1) (C) values tended to increase as fT4 increased.

As a result of analyzing fT4 by dividing it by deciles, the obstructive lung pattern (p = 0.018), FVC (L) (p<0.001) and FEV1 (L) (p = 0.002) values ​​tended to increase as fT4 increased (Fig 2).

The results of logistic regression analyses for the risk of obstructive lung pattern by fT4 quartiles are shown in Table 3.

**Table 3:** Odds ratios (ORs) and 95% confidence intervals (CIs) for obstructive lung pattern based on free thyroxine quartiles.

	fT4 quartile			
	Q1 (0.89–1.09 ng/dL) (n = 294, 23.4%)	Q2 (1.10–1.19 ng/dL) (n = 321, 25.5%)	Q3 (1.20–1.30 ng/dL) (n = 317, 25.2%)	Q4 (1.31–1.76 ng/dL) (n = 329, 25.9%)
**Obstructive pattern**				
**Model 1** **Model 2** **Model 3** **Model 4**	1.000 1.000 1.000 1.000	1.984 (0.864, 4.556) 1.944 (0.819, 4.614) 2.041 (0.817, 5.095) 1.998 (0.797, 5.011)	2.898 (1.326, 6.334) † 2.777 (1.246, 6.189) † 2.818 (1.243, 6.389) † 2.875 (1.265, 6.535) †	3.664 (1.731, 7.754) * 2.878 (1.322, 6.268) † 3.000 (1.340, 6.716) † 2.970 (1.287, 6.854) †

fT4, free thyroxine.

Model 1, unadjusted; Model 2, with adjustment for age and sex; Model 3 as model 2, with additional adjustment for occupation, smoking, alcohol consumption, walking activity, and body mass index; Model 4 as model 3, with additional adjustment for glycated hemoglobin, and thyroid-stimulating hormone.

OR and 95% CI for risk of obstructive lung pattern were estimated using logistic regression models. All p values and 95% CI for OR were corrected by Bonferroni’s method due to multiple testing.

*p<0.001,

†0.001≤p<0.01.

Subjects in higher fT4 quartiles (Q3 and Q4) had a significantly greater risk of obstructive lung pattern than those in the lowest fT4 quartile (Q1) (for Q3, OR 2.898, 95% CI 1.326–6.334, p = 0.003; for Q4, OR 3.664, 95% CI 1.731–7.754, p<0.001). With the Q1 group as reference, the multivariate-adjusted ORs (95% CIs) for obstructive lung pattern in the Q3 and Q4 groups were 2.875 (1.265–6.535) and 2.970 (1.287–6.854), respectively.

## Discussion

In this cross-sectional study of 1,261 euthyroid middle-aged subjects based on data from the KNHANES VI, high fT4 independently predicted obstructive lung pattern and was associated with an increased probability of obstructive lung pattern after adjusting for age, sex, occupation, smoking, alcohol consumption, walking activity, body mass index, glycated hemoglobin and TSH. To the best of our knowledge, this is the first study to report an association between thyroid hormones and lung function in a generally healthy population.

Several small-patient studies have attempted to link thyroid function with lung function in patients with COPD. Gow *et al*. reported that there were no differences in fT4 and TSH levels between patients with COPD and controls [14]. A study by Okutan *et al*. demonstrated that free triiodothyronine levels were lower in COPD groups than in controls [16]. However, the authors also did not find any differences in fT4 and TSH levels between the COPD group and healthy control group. A recent study by Terzano *et al*. found a negative relationship between partial pressure of oxygen and TSH levels in patients with COPD [7]. However, they observed no significant differences in lung function between COPD patients with thyroid disease and controls. Dimopoulou *et al*. evaluated 46 COPD patients with euthyroidism and found a strong positive association between total triiodothyroine/total thyroxine ratio and partial pressure of oxygen. However, no correlation was found between thyroid hormone levels and spirometry parameters in the patients with COPD [15]. Further, they did not include controls. These inconsistent findings [7,14–16] may possibly be due to the small number of cases and differences in the severity of COPD.

Cigarette smoking is the key environmental risk factor for COPD and causes not only airway and lung inflammation but also systemic cellular and humoral inflammation, and oxidative stress [13]. Some studies [19,28,29], including a study [19] in the same cohort as ours, have shown that smoking is significantly associated with low serum TSH levels. Vanderver *et al*. reported that hypothyroxinemia was smoking among women with high iodine intakes [30], while Kadkhodazadeh *et al*. demonstated that fT4 levels was higher in current smokers compared to former and never smokers [29], consistent with our findings (S1 Table). Because smoking can affect both obstructive lung pattern and thyroid function, we adjusted for smoking status (current heavy, current moderate, current light, former and never smokers) to investigate the association between thyroid hormones and obstructive lung pattern. Subjects with obstructive lung pattern showed lower levels of unadjusted TSH than those with normal lung function. However, there was no significant difference between the groups in smoking-adjusted mean TSH levels. Few studies have investigated the relationship between thyroid function and lung function in the general population. Ittertmann *et al*. pooled data from two independent population-based studies and found no consistent associations between thyroid function and lung function [9]. They assessed thyroid function using TSH, but not fT4. Serum TSH levels within the reference range were negatively associated with FEV_1_/FVC, but there were no significant differences in FEV_1_/FVC between the third quintile and the other TSH quintiles [9]. In the present study, spirometry parameters of FVC, FEV_1_, FEV_1_/FVC, and FEF_25-75_ were not significantly different among the TSH quartile groups (S2 Table), consistent with previous findings [9]. Meanwhile, subjects with obstructive lung pattern had higher unadjusted and smoking-adjusted fT4 levels than those with normal lung function. Furthermore, a positive association between fT4 quartiles and the proportion of obstructive lung pattern was observed. The hazard ratio for obstructive lung pattern increased from the lowest to the highest quartiles of fT4 even after adjustment for confounders.

Although the pathophysiological mechanism underlying the association between high fT4 levels and obstructive lung pattern remains to be established, the potential mechanisms are adaptation process, protective effect and systemic inflammation. Thyroid hormones are critical determinants of energy homeostasis and metabolism (9), and COPD patients may need to exert more effort to breathe, thus increasing metabolism (10). Further, the moderate increase in fT4 levels within the upper normal range in subjects with obstructive lung pattern may be regarded as an adaptation strategy for the increased energy expenditure and metabolism resulting from the higher respiratory effort to meet the oxygen demand. High levels of circulating thyroid hormones in hyperthyroid mice blocked myosin light chain phosphorylation, resulting in a tighter lung barrier or protective conditions against lung injury [31]. Further, these high thyroid hormone levels attenuated lung injury by modulating expression of the claudin 4 proteins involved in junctional tightness [32]. Another in vitro analysis showed that thyroid hormone markedly increased airway smooth muscle proliferation in the presence of transforming growth factor beta 1 [33], suggesting thyroid hormone-induced airway remodeling. Therefore, it could be considered that thyroid hormones are increased as a protective effect or a repair process against lung injury in patients with obstructive lung pattern. In contrast, COPD can be seen as a pulmonary component of a chronic systemic inflammatory syndrome [13]. A recent prospective study in healthy middle-aged subjects showed that elevated inflammatory markers were closely associated with an increased risk of future impairment of obstructive lung pattern (FEV_1_/FVC ≤0.7) [34]. Thus, systemic inflammatory responses in individuals with obstructive lung pattern may affect thyroid function. In addition, comorbid conditions, such as cardiovascular disease, diabetes and hypertension, are highly prevalent among patients with COPD [12]. These comorbidities may partially explain the risk of obstructive lung pattern in high fT4 levels. However, there is limited evidence concerning the relationship between obstructive lung pattern and thyroid function, and thus, further research on the potential mechanisms is needed.

To date, no study on the association between fT4 levels and obstructive lung patterns has been published. To our best knowledge, our study is the first to determine the relationship between thyroid function and lung function in euthyroid subjects based on a nationally representative population in Korea. We were able to control the potentially confounding effects of risk factors for obstructive lung pattern, including occupation, smoking, alcohol consumption, physical activity, thyroid dysfunction and additional comorbid conditions, in examining the relationship between thyroid function and lung function. However, owing to its cross-sectional nature, we could not determine the causal relationship between thyroid hormone levels and lung function. Additional prospective, high-quality, randomized controlled trials are needed to further clarify the relationship between fT4 and obstructive lung pattern.

In conclusion, a high fT4 level is an independent predictor of obstructive lung pattern in euthyroid middle-aged subjects. Further prospective studies are needed to confirm these findings.

## Supporting information

S1 Table Comparison of thyroid hormone levels according to smoking status. TSH, thyroid-stimulating hormone; fT4, free thyroxine. Data are presented as means (standard error). TSH and fT4 levels according to smoking status were compared using general linear model.
(DOCX)

S2 Table Comparison of spirometry data according to thyroid-stimulating hormone quartiles. TSH, thyroid-stimulating hormone; FVC, forced vital capacity; FEV_1_, forced expiratory volume in one second; FEF_25-75_, forced expiratory flow, mid-expiratory phase. Data are presented as means (standard error) or weighted percentages as appropriate for the variable. Spirometry data of the study population with respect to the thyroid-stimulating hormone quartiles were compared using general linear model for continuous variables and chi-square test for categorical variables.
(DOCX)

## References

[1] Braverman LE, Cooper DS, Kopp P. Werner & Ingbar’s The Thyroid: A Fundamental and Clinical Text. Philadelphia, PA Wolters Kluwer Health; 2020.

[2] Jameson JL, Fauci AS, Kasper DL, Hauser SL, Longo DL, Loscalzo J. Harrison’s Principles of Internal Medicine, 20e. New York, NY: McGraw-Hill Education; 2018.

[3] Goswami R, Guleria R, Gupta AK, Gupta N, Marwaha RK, Pande JN, et al. Prevalence of diaphragmatic muscle weakness and dyspnoea in Graves’ disease and their reversibility with carbimazole therapy. Eur J Endocrinol. 2002; 147(3):299–303. 10.1530/eje.0.1470299 . 12213666

[4] Kahaly GJ, Nieswandt J, Wagner S, Schlegel J, Mohr-Kahaly S, Hommel G. Ineffective cardiorespiratory function in hyperthyroidism. J Clin Endocrinol Metab. 1998; 83(11):4075–4078. 10.1210/jcem.83.11.5275 . 9814494

[5] Ladenson PW, Goldenheim PD, Ridgway EC. Prediction and reversal of blunted ventilatory responsiveness in patients with hypothyroidism. Am J Med. 1988; 84(5):877–883. 10.1016/0002-9343(88)90066-6 . 3364447

[6] Ambrosino N, Pacini F, Paggiaro PL, Martino E, Contini V, Turini L, et al. Impaired ventilatory drive in short-term primary hypothyroidism and its reversal by L-triiodothyronine. J Endocrinol Invest. 1985; 8(6):533–536. 10.1007/bf03348555 . 3833897

[7] Terzano C, Romani S, Paone G, Conti V, Oriolo F. COPD and thyroid dysfunctions. Lung. 2014; 192(1):103–109. 10.1007/s00408-013-9537-6 . 24281671

[8] Birring SS, Patel RB, Parker D, McKenna S, Hargadon B, Monteiro WR, et al. Airway function and markers of airway inflammation in patients with treated hypothyroidism. Thorax. 2005; 60(3):249–253. 10.1136/thx.2004.034900 . 15741445 PMC1747336

[9] Ittermann T, Gläser S, Ewert R, Felix S, Völzke H, Dörr M. Serum thyroid-stimulating hormone levels are not associated with exercise capacity and lung function parameters in two population-based studies. BMC Pulm Med. 2014; 14:145. 10.1186/1471-2466-14-145 . 25182209 PMC4236747

[10] Godfrey MS, Jankowich MD. The Vital Capacity Is Vital: Epidemiology and Clinical Significance of the Restrictive Spirometry Pattern. Chest. 2016; 149(1):238–251. 10.1378/chest.15-1045 . 26356330

[11] Vogelmeier CF, Criner GJ, Martinez FJ, Anzueto A, Barnes PJ, Bourbeau J, et al. Global Strategy for the Diagnosis, Management, and Prevention of Chronic Obstructive Lung Disease 2017 Report. GOLD Executive Summary. Am J Respir Crit Care Med. 2017; 195(5):557–582. 10.1164/rccm.201701-0218PP . 28128970

[12] Mannino DM, Thorn D, Swensen A, Holguin F. Prevalence and outcomes of diabetes, hypertension and cardiovascular disease in COPD. Eur Respir J. 2008; 32(4):962–969. 10.1183/09031936.00012408 . 18579551

[13] Fabbri LM, Rabe KF. From COPD to chronic systemic inflammatory syndrome? Lancet. 2007; 370(9589):797–799. 10.1016/s0140-6736(07)61383-x . 17765529

[14] Gow SM, Seth J, Beckett GJ, Douglas G. Thyroid function and endocrine abnormalities in elderly patients with severe chronic obstructive lung disease. Thorax. 1987; 42(7):520–525. 10.1136/thx.42.7.520 . 3125626 PMC460817

[15] Dimopoulou I, Ilias I, Mastorakos G, Mantzos E, Roussos C, Koutras DA. Effects of severity of chronic obstructive pulmonary disease on thyroid function. Metabolism. 2001; 50(12):1397–1401. 10.1053/meta.2001.28157 . 11735083

[16] Okutan O, Kartaloglu Z, Onde ME, Bozkanat E, Kunter E. Pulmonary function tests and thyroid hormone concentrations in patients with chronic obstructive pulmonary disease. Med Princ Pract. 2004; 13(3):126–128. 10.1159/000076950 . 15073423

[17] Kim WG, Kim WB, Woo G, Kim H, Cho Y, Kim TY, et al. Thyroid Stimulating Hormone Reference Range and Prevalence of Thyroid Dysfunction in the Korean Population: Korea National Health and Nutrition Examination Survey 2013 to 2015. Endocrinol Metab (Seoul). 2017; 32(1):106–114. 10.3803/EnM.2017.32.1.106 . 28116874 PMC5368108

[18] Choi CJ, Seo M, Choi WS, Kim KS, Youn SA, Lindsey T, et al. Relationship between serum 25-hydroxyvitamin D and lung function among Korean adults in Korea National Health and Nutrition Examination Survey (KNHANES), 2008–2010. J Clin Endocrinol Metab. 2013; 98(4):1703–1710. 10.1210/jc.2012-3901 . 23533242

[19] Park S, Kim WG, Jeon MJ, Kim M, Oh HS, Han M, et al. Serum thyroid-stimulating hormone levels and smoking status: Data from the Korean National Health and Nutrition Examination Survey VI. Clin Endocrinol (Oxf). 2018; 88(6):969–976. 10.1111/cen.13606 . 29604104

[20] Kwon HM, Yang IH, Park KK, Cho BW, Byun J, Lee WS. Cigarette smoking and knee osteoarthritis in the elderly: Data from the Korean National Health and Nutritional Examination Survey. Exp Gerontol. 2020; 133:110873. 10.1016/j.exger.2020.110873 . 32044381

[21] Sanyal AJ, Brunt EM, Kleiner DE, Kowdley KV, Chalasani N, Lavine JE, et al. Endpoints and clinical trial design for nonalcoholic steatohepatitis. Hepatology. 2011; 54(1):344–353. 10.1002/hep.24376 . 21520200 PMC4014460

[22] Lee YH, Kim SU, Song K, Park JY, Kim DY, Ahn SH, et al. Sarcopenia is associated with significant liver fibrosis independently of obesity and insulin resistance in nonalcoholic fatty liver disease: Nationwide surveys (KNHANES 2008–2011). Hepatology. 2016; 63(3):776–786. 10.1002/hep.28376 . 26638128

[23] Kim HJ, Park SJ, Park HK, Byun DW, Suh K, Yoo MH. Thyroid autoimmunity and metabolic syndrome: a nationwide population-based study. Eur J Endocrinol. 2021; 185(5):707–715. 10.1530/eje-21-0634 . 34519275

[24] Jang J, Kim Y, Shin J, Lee SA, Choi Y, Park EC. Association between thyroid hormones and the components of metabolic syndrome. BMC Endocr Disord. 2018; 18(1):29. 10.1186/s12902-018-0256-0 . 29783969 PMC5963056

[25] Miller MR, Hankinson J, Brusasco V, Burgos F, Casaburi R, Coates A, et al. Standardisation of spirometry. Eur Respir J. 2005; 26(2):319–338. 10.1183/09031936.05.00034805 . 16055882

[26] Lee SW. Methods for testing statistical differences between groups in medical research: statistical standard and guideline of Life Cycle Committee. Life Cycle. 2022; 2:e1. 10.54724/lc.2022.e1.

[27] Lee SW. Regression analysis for continuous independent variables in medical research: statistical standard and guideline of Life Cycle Committee. Life Cycle. 2022; 2:e3. 10.54724/lc.2022.e3.

[28] Belin RM, Astor BC, Powe NR, Ladenson PW. Smoke exposure is associated with a lower prevalence of serum thyroid autoantibodies and thyrotropin concentration elevation and a higher prevalence of mild thyrotropin concentration suppression in the third National Health and Nutrition Examination Survey (NHANES III). J Clin Endocrinol Metab. 2004; 89(12):6077–6086. 10.1210/jc.2004-0431 . 15579761

[29] Kadkhodazadeh H, Amouzegar A, Mehran L, Gharibzadeh S, Azizi F, Tohidi M. Smoking status and changes in thyroid-stimulating hormone and free thyroxine levels during a decade of follow-up: The Tehran thyroid study. Caspian J Intern Med. 2020; 11(1):47–52. 10.22088/cjim.11.1.47 . 32042386 PMC6992726

[30] Vanderver GB, Engel A, Lamm S. Cigarette smoking and iodine as hypothyroxinemic stressors in U.S. women of childbearing age: a NHANES III analysis. Thyroid. 2007; 17(8):741–746. 10.1089/thy.2006.0332 . 17725431

[31] Pajouhi N, Owji M, Naghibalhossaini F, Omrani GH, Varedi M. Modulation by thyroid hormone of myosin light chain phosphorylation and aquaporin 5 protein expression in intact lung. J Physiol Biochem. 2015; 71(1):99–106. 10.1007/s13105-015-0386-z . 25649359

[32] Varedi M, Pajouhi N, Owji M, Naghibalhossaini F, Omrani GHR. Differential modulation of claudin 4 expression and myosin light chain phosphorylation by thyroid function in lung injury. Clin Respir J. 2017; 11(6):797–804. 10.1111/crj.12418 . 26619308

[33] Dekkers BG, Naeimi S, Bos IS, Menzen MH, Halayko AJ, Hashjin GS, et al. L-thyroxine promotes a proliferative airway smooth muscle phenotype in the presence of TGF-β1. Am J Physiol Lung Cell Mol Physiol. 2015; 308(3):L301–306. 10.1152/ajplung.00071.2014 . 25480330

[34] Kalhan R, Tran BT, Colangelo LA, Rosenberg SR, Liu K, Thyagarajan B, et al. Systemic inflammation in young adults is associated with abnormal lung function in middle age. PLoS One. 2010; 5(7):e11431. 10.1371/journal.pone.0011431 . 20625390 PMC2896391

