# Supplementary material for: Association of free thyroxine with obstructive lung pattern in euthyroid middle-aged subjects: A population-based study
Source: PLoS One. 2022 Jul 22;17(7):e0270126. doi: 10.1371/journal.pone.0270126 (PMC9307150; doi:10.1371/journal.pone.0270126)
Supplement: S1 Table — TSH, thyroid-stimulating hormone; fT4, free thyroxine. Data are presented as means (standard error). TSH and fT4 levels according to smoking status were compared using general linear model. (DOCX) [file pone.0270126.s001.docx]

| Variables | TSH (mIU/L) | p value | fT4 (ng/dL) | p value |
| --- | --- | --- | --- | --- |
| Current  Light  Moderate  Heavy | 2.09 (0.07)  2.28 (0.14)  1.94 (0.77)  2.30 (0.24) | <0.001 | 1.23 (0.01)  1.22 (0.02)  1.23 (0.01)  1.27 (0.04) | <0.001 |
| Former | 2.45 (0.07) |  | 1.23 (0.01) |  |
| Never | 2.64 (0.05) |  | 1.18 (0.01) |  |
