# Supplementary material for: Association of free thyroxine with obstructive lung pattern in euthyroid middle-aged subjects: A population-based study
Source: PLoS One. 2022 Jul 22;17(7):e0270126. doi: 10.1371/journal.pone.0270126 (PMC9307150; doi:10.1371/journal.pone.0270126)
Supplement: S2 Table — TSH, thyroid-stimulating hormone; FVC, forced vital capacity; FEV1, forced expiratory volume in one second; FEF25-75, forced expiratory flow, mid-expiratory phase. Data are presented as means (standard error) or weighted percentages as appropriate for the variable. Spirometry data of the study population with respect to the thyroid-stimulating hormone quartiles were compared using general linear model for continuous variables and chi-square test for categorical variables. (DOCX) [file pone.0270126.s002.docx]

| Variables | TSH quartile | | | | p value |
| --- | --- | --- | --- | --- | --- |
|  | **Q1**  **(0.62-1.56 mIU/L)**  **(n=314, 25.1%)** | **Q2**  **(1.57-2.23 mIU/L)**  **(n=317, 25.8%)** | **Q3**  **(2.24-3.11 mIU/L)**  **(n=316, 24.8%)** | **Q4**  **(3.11-6.68 mIU/L)**  **(n=314, 24.3%)** |  |
| Spirometry parameters |  |  |  |  |  |
| FVC (%, predicted) | 95.59 (0.63) | 95.42 (0.57) | 95.68 (0.63) | 95.87 (0.62) | 0.965 |
| FEV_1_ (%, predicted) | 93.50 (0.72) | 93.99 (0.55) | 93.95 (0.68) | 94.68 (0.69) | 0.707 |
| FEV_1_/FVC | 0.775 (0.004) | 0.779 (0.004) | 0.773 (0.004) | 0.781 (0.004) | 0.435 |
| FEF_25-75_ (L/sec) | 2.83 (0.07) | 2.80 (0.05) | 2.69 (0.05) | 2.75 (0.06) | 0.338 |
| Obstructive lung pattern (%) | 12.4% | 11.1% | 11.3% | 8.7% | 0.618 |
